# Supplementary material for: Examining delays in diagnosis for slipped capital femoral epiphysis from a health disparities perspective
Source: PLoS One. 2022 Jun 24;17(6):e0269745. doi: 10.1371/journal.pone.0269745 (PMC9231816; doi:10.1371/journal.pone.0269745)
Supplement: Attachment — Submitted filename: Response to Reviewers.docx [file pone.0269745.s001.docx]

Center for Anatomical Science

April 26, 2022

Rebuttal Letter

Dear PLOS ONE Academic Editor and Reviewers:

First, I would like to thank the Editors at PLOS ONE for allowing us to revise our manuscript based on the reviewers concerns and comments. Second, we want to say thanks to the reviewers for their time spent in review of this manuscript and the suggestions made to make the manuscript better.

We understand that a submission requirement for this journal is to provide a copy of the dataset [for whatever purposes]. However, in order for this study to be possible, the authors had to enter into a data user agreement with the Institutional Review Board (IRB) at Cooks Children’s Healthcare System where data were collected, wherein the site retains all rights and access to these data. Therefore, although the authors are not allowed to include the data, we are happy to provide the appropriate staff with the contact information for the IRB noted above, so that the journal may communicate with the IRB and obtain a copy of these data.

The phone number for the Institutional Review Board in the Grants Office is 682-885-2103. Email: [CookChildrenResearch@cookchildrens.org](mailto:CookChildrenResearch@cookchildrens.org)

From the reviewers’ feedback, questions, comments, here are our responses.

**Reviewer #1:**

In the letter from Dr. Kuman, there were two number 1 comments, so we split them up into 1A and 1B.

Comment 1A. Change terminology from “physician” to primary care provider or healthcare provider throughout the manuscript to be more inclusive of other healthcare providers such as advanced practice registered nurses (nurse practitioners) and physician assistants.

Response 1A. For their first comment, we thought this was a great recommendation and changed any reference throughout the entire manuscript from “physician” to “primary care provider”. We also changed the wording in Figure 2.

Comment 1B. There are minor grammatical errors throughout the document that need to be reviewed and revised.

Response 1B. We carefully reviewed the manuscript for grammatical errors. In addition, we solicited outside help with these corrections.

Comment 2. In the introduction section this question should be answered: Why are black and Hispanic children more likely to be diagnosed with SCFE? It will be important to include this information for readers who are not as well versed in SCFE and are reading this manuscript because of their interest in the impact of health disparities on healthcare outcomes. It also reinforces the information listed in lines 197-200 and 243.

Response 2. In the introduction, we added some additional content indicating why different ethnic groups are more often diagnosed with SCFE (See Lines 69-71, Page 3-4). Currently, there is no consensus among researchers exactly why a higher incident in minorities exists, but theories around larger body size and skeletal morphology might explain the differences. In addition, only a few studies with a sizeable Hispanic patient size have occurred to address this issue.

Comment 3. Can the first sentence of the discussion section be added to the introduction as part of the purpose of the study?

Response 3. We agree with this suggestion. The first sentence of the discussion section was added to the introduction as part of the purpose of the study. See Lines 81-82 on Page 4.

Comment 4. I really appreciate how the authors included why the location of this study (Texas) was important to include in the manuscript.

Response 4. We agree. Texas has such a large Hispanic population, and we did feel it was important to share the location of the study with the readers. We discuss potential barriers for care in the state on Line 223-225, Page 11. In addition, we discuss Texas’ rate of uninsured children in Lines 236-238, Page 11.

Comment 5. Starting with line 218, I wanted the authors to provide examples of the barriers to care that were referred to. However, I realized that the examples were in the next paragraph. For continuity of content/flow of content, I recommend either combining the paragraph that starts with line 222 to the preceding paragraph or consider at sentence at the end of the end of

line 221 to prepare readers for the barriers that you’re going to discuss in the upcoming paragraphs.

Response 5. After reading those paragraphs on line 218 (original manuscript) in the Discussion, we agree with the reviewer. We combined those two paragraphs which are now the 2^nd^ paragraph of the Discussion of the revised manuscript. See Lines 221-228 where those two paragraphs were combined.

Comment 6. In line 240, can you make the connection to health disparities?

Response 6. This did need to be discussed in more detail. Beginning on Line 275, Page 13 we address this concern by adding information about how Cook Childrens Healthcare System employs a “medical home” model in their neighborhood clinics.

Comment 7. Lines 258-259 “Healthcare providers can also carry unfavorable bias toward obese patients” resulting in? (Consider adding more information that highlights the impact of obesity bias on healthcare outcomes)

Response 7. We have added more information regarding “obese patients” on Lines 286-292 Page 13-14 to emphasize bias by healthcare providers.

Comment 8. I do not understand the relevance of including line 84 if this is a standard course of action. Including this line may also prompt readers to wonder why there was one exception. I recommend removing line 84.

Response 8. At the advice of the reviewer, we removed line 84, and agree it was not relevant.

Comment 9. Table 2 is very well constructed/easy to follow and understand

Response 9. We appreciate the reviewer’s positive comment about Table 2. We will leave as is!

Comment 10. I appreciate the information that was included in lines 268-271

Response 10. Again, we appreciate the positive comment, and that information was left in the manuscript.

Comment 11. In line 275, are there any other possible reasons that patients who are uninsured or covered by Medicaid may be diagnosed in emergency departments that can be supported by the literature?

Response 11. In Lines 258-262, Page 12, we address possible reasons to explain why patients who are insured or covered with Medicaid presented at emergency departments for diagnosis that were supported by the literature. This could be based on transportation difficulties, work schedule conflicts, and barriers obtaining appointments with primary care or specialists (orthopedic) clinics.

Comment 12. Overall, very well written discussion section that highlights key points from the results.

Response 12. We appreciate the comments about the Discussion section. We have made a few changes based on Reviewer #2 suggestions making it more focused.

Comment 13. Succinct abstract, well-written. Highlights key points

Response 13. Again, thanks for the positive comments!

Comment 14. Although this may drastically increase your word count, I still recommend the use of “people-first language” throughout the manuscript. For example, instead of “Medicaid patients,” consider changing to patients insured by Medicaid. There are also several other opportunities to include people first language throughout the manuscript.

Response 14. Great suggestion that makes the reading of the manuscript much stronger. We went through the manuscript and changed to “people-first-language” as suggested.

**Reviewer #2:**

Comment: In this study, authors examining delays in diagnosis for slipped capital femoral epiphysis from a health disparities perspective. 1) Proofreading the manuscript is a must to avoid errors in writing. 2) Ensure mention of the limitations of the study within discussion, along with future directions. 3) The discussion can be improved and more centered.

Comment 1: Proofreading the manuscript is a must to avoid errors in writing.

Response 1: We have made an extensive effort to proofread the document and correct any writing or grammatical errors, including a 3^rd^ review for additional editing.

Comment 2: Ensure mention of the limitations of the study within discussion, along with future directions.

Response 2: We have added a section in the discussion with the limitations (2^nd^ to last paragraph, Line 297) and future directions of the study (last paragraph, Line 309).

Comment 3: The discussion can be improved and more centered.

Response 3: We have improved upon the content and focus of the discussion.

We appreciate the comments and concerns from both reviewers. Thank you for your time and assistance with reviewing the manuscript. In addition to the reviewer’s comments/concerns, we placed the two tables back-to-back in the manuscript, and then slightly changed the order of the figures. Every figure/table was kept the exact same as well as the results for the tables/figures; however, we did feel the order they are in now makes it easier for the reader to follow. Again, we are grateful for the extremely thorough review of our manuscript and thankful for the time and energy put in by the reviewers and editorial team to support this study.

Sincerely,

Rustin E. Reeves, PhD

Professor and Director

Center for Anatomical Sciences

UNT Health Science Center

3500 Camp Bowie Blvd.

Fort Worth, TX 76107
